# Editorial: Imaginative culture and human nature: Evolutionary perspectives on the arts, religion, and ideology

**DOI:** 10.3389/fpsyg.2022.999057

**Published:** 2022-08-31

**Authors:** Joseph Carroll, John A. Johnson, Emelie Jonsson, Rex E. Jung, Valerie van Mulukom

**Affiliations:** ^1^Department of English, University of Missouri–St. Louis, St. Louis, MO, United States; ^2^Department of Psychology, The Pennsylvania State University (PSU), State College, PA, United States; ^3^Department of English, University of Tromsø, Tromsø, Norway; ^4^Department of Psychology, University of New Mexico, Albuquerque, NM, United States; ^5^Centre for Trust, Peace, and Social Relations, Coventry University, Coventry, United Kingdom

**Keywords:** imagination, culture, evolution, religion, ideology, art, literature, music

## Imagination

In the overview describing this Research Topic and soliciting participants, we said that the Research Topic encompassed four main areas in the evolutionary human sciences: evolutionary behavioral science, gene-culture coevolution, emotions, and “cognitive neuroscience, which has identified the Default Mode Network as the central neurological location of the human imagination.” The 20 articles accepted for publication fulfilled our expectations for the first three of these Research Topics. All the articles make extensive reference to research in the evolutionary behavioral sciences. Many of the articles discuss the evolved cognitive dispositions that make cumulative culture possible. Several articles make emotions a salient part of their arguments. Only one article gives serious attention to the Default Mode Network (DMN) (Newberg et al., “Orgasmic Meditation”). Over the past two decades, a rapidly expanding body of research has revealed that the DMN makes it possible to abstract away from the immediate present, to think about past and future, to enter into other minds, and to construct fictional worlds (Buckner and DiNicola, 2019; Carroll, 2020; van Mulukom, 2020).

Though not referencing the DMN, several articles in this Research Topic invoke its functions. The defining characteristic of the DMN is the brain's ability to turn inward to mentally generated representations decoupled from the immediate external environment. Kapitany et al. point toward “counterfactual thinking,” the “ability to imagine things that are not real,” and the ability to construct a “fictional reality” (“Pretensive Shared Reality”). Yang et al. focus on pretend or imaginary domains (“Why iPlay”). Teasdale et al. invoke the “capacity to step back from the immediacy of the present world to consider an alternative parallel world (whether fictional or a real world elsewhere in space or time)” (“Drama”). Flinn says that we humans have “a theater in our minds” (“The Creative Neurons”). Steen and Chakraborty describe the imagination building “scenarios” of possible future states (“Exploring the Possible”). Several articles focus not just on imagination but on shared or collective imagination (Lauer, “Language, Childhood, and Fire”). Three articles work out mathematical models for analyzing the transmission of cultural artifacts across populations (Ganesh and Gabora, “Discontinuous Cultural Evolution,” Martins and Baumard, “Reliable Instruments,” and Tran et al., “Kolam Art”).

## The adaptive functions of imaginative culture

All the articles in this Research Topic agree that imaginative activity is a pervasive feature of human life, but they do not agree on whether it is adaptive, or if adaptive, what its adaptive functions are. Dubourg and Baumard argue that creating narrative fictions is a byproduct of other adaptive cognitive capacities but also identify various secondary adaptive functions narrative fictions could serve: signaling mate value, gaining social status, transmitting knowledge, communicating social norms, or selling products (“Entertainment Technologies”). All those functions can be attributed to forms of imaginative activity other than creating narrative fictions, and the list of possible functions can be expanded. Mendoza Straffon isolates and emphasizes gaining social status (“The Peacock Fallacy”). Larsen examines the functional effects of internalized religious norms in Scandinavian social democracy (“The Lutheran Imaginary”). Steen and Chakraborty argue that the imagination functions adaptively for individuals by building scenarios to test possible future courses of action. Flinn argues for a group-level adaptive function: the ability of “collective imaginations” to generate innovations. McCrae discusses various hypotheses for the adaptive function of music and supports Perlovsky's idea that “that music co-evolved with language to compensate for the hypertrophy of cognition that language facilitated.... Music and musical emotion evolved because it restores the unity of the self and thus the will to live” (“Music Lessons”). Gabriel suggests a similar existential function: the arts equip us with “a motivated understanding of ourselves” (“Affect, Belief, and the Arts”).

The existential functions suggested by McCrae and Gabriel can be generalized beyond the arts or any specific art. If we identify the imagination with the DMN, we can characterize the adaptive function of the imagination as an activity of synthesis. In “The Creative Neurons,” Flinn says, “It seems unlikely that there are singular ‘creative neurons' or even localized modules for imagination; these abilities instead result from complex systems involving interaction among many parts of the brain.” The DMN is the central organizing network for those interactions. In their neuroimaging study of orgasmic meditation, Newberg et al. describe some of the neural regions that coactivate within the DMN. “The DMN is characterized by the synchronous activation of several separated regions in the brain, including the medial prefrontal cortex, posterior cingulate cortex, precuneus, inferior parietal lobule, and inferolateral temporal cortex.” The DMN is the most widely connected network in the brain. It integrates information from diverse sources and uses that information to construct an emotionally and aesthetically modulated model of the world (Margulies et al., 2016; Buckner and DiNicola, 2019). What Flinn calls a “theater in the mind” is a simulation, a virtual reality, that directs human behavior. As McCrae and Gabriel contend, the emotional and aesthetic modulations in imaginative constructs provide people with a sense of “meaning.”

## The scope of Research Topics

Articles in this Research Topic take in macro-narratives of religion and ideology, intimate personal relationships, and self-images in individuals. Gabriel focuses on religion, Larsen and Martins and Baumard on ideology. Newberg et al. discuss a meditative practice that involves a male stimulating a partner's clitoris. Yang et al. discuss sex differences in video-gaming. Luoto mines literary texts for differences in gendered linguistic usage (“Sexual Dimorphism”). McCrae concentrates on qualities of emotion in the individuals who create and listen to music. Barrett discusses the emotional richness of experience provided by all forms of imaginative culture (“Positive Experience”).

Articles discussing the arts encompass music (McCrae), the verbal arts (Dubourg and Baumard; Lauer; Luoto; Martins and Baumard; Teasdale et al.), the visual arts (Coss and Charles, “Prehistoric Art,” Tran et al.), and dance (Dobrowolski and Pezdek, “Movement”). Kozbelt discusses multiple arts and identifies a host of cross-modal evolved cognitive dispositions that help to explain the formal organization of specific arts and the way the arts change over historical time (“The Aesthetic Legacy of Evolution”). Multiple arts are also mentioned by Gabriel, Ganesh and Gabora, and Steen and Chakraborty.

## Disciplinary orientation

Contributors to this Research Topic include psychologists, anthropologists, biologists, neuroscientists, philosophers, art historians, media specialists, literary scholars, and experts in physical education. Eight of the 20 articles are empirical studies (Coss and Charles; Ganesh and Gabora; Kapitany et al.; Luoto, Newberg et al.; Teasdale et al.; Tran et al.; Yang et al.). Three articles offer innovations in mathematical modeling of cultural transmission (Martins and Baumard). The remaining articles are classified in *Frontiers in Psychology* as “conceptual analysis,” “hypothesis and theory,” and “opinion.”

All the articles cite research from the evolutionary human sciences. One can nonetheless distinguish between articles for which evolutionary biology provides a conceptual matrix and those for which some speculative philosophical system provides the matrix. Barrett's frame of reference is primarily in philosophy, that of Dobrowolski and Pezdek in phenomenology (Husserl and Heidegger) and Lacanian psychoanalysis.

Rough distinctions can be drawn among three groups of articles: (a) those that use the evolutionary human sciences to study some particular kind of cultural artifact (Lauer; Larsen; Kapitany et al.; Newberg et al.; Tran et al.); (b) those that use cultural artifacts to study some particular feature of human nature or human behavior (Coss and Charles; Ganesh and Gabora; Luoto; Martins and Baumard; Teasdale et al.; Yang et al.); and (c) those that construct some general theory about the psychological and/or anthropological basis of imaginative culture (Barrett; Dubourg and Baumard; Dobrowolski and Pezdek; Flinn; Gabriel; Mendoza Straffon; Steen and Chakraborty). Two articles, those by Kozbelt and McCrae, could be placed in both of the first two categories. They aim to use the evolutionary human sciences to further their understanding of music (McCrae) or of the arts in general (Kozbelt), and they also aim to use the arts to improve their knowledge of human psychology.

We shall give just one example each of articles that more unambiguously exemplify the three categories. Kapitany et al. cite much research on childhood play and on adult pretend play, but they are aiming less at altering the structure of knowledge in these fields than in using these fields to illuminate one particular kind of cultural practice—adult participation in *Dungeons and Dragons* (a fantasy role-playing game). Luoto, in contrast, is using large databases of digitized literary works to examine sex differences in linguistic usage. The focus is not on the literature itself but on evolved sex differences. Flinn can serve to illustrate the third category. He focuses on no one particular kind of cultural artifact or cultural practice. He is concerned rather to construct a conceptual model of human life history phases and relationships and to explain how human reproductive and social practices generate cumulative culture.

## Basic concepts in imaginative culture

Several of the articles can be used to illuminate basic concepts in imaginative culture. Flinn lodges the evolved systemic logic of human life history and social organization in fundamental principles of reproduction and natural selection. He also makes it clear that imaginative culture is an integral part of that systemic logic. Lauer and Kapitany et al. emphasize the way the specifically human life history feature of extended childhood has evolved to accommodate the peculiarly human needs of imagination. Gabriel evokes the pervasive presence of imaginative culture in all aspects of human life, from the most individual need for purpose and meaning to the religious rituals in which individuals are submerged within a collective social identity. By concentrating on a single form of public folk art in a South Indian community, Tran et al. give readers a concrete feeling for the way the arts are both intimately personal and socially communicative. McCrae and Kozbelt cogently demonstrate that the finest, most subtle aspects of artistic form and meaning can be explained by evolved affective and cognitive features of the human mind. Larsen extends an evolutionary conception of human nature to complex historical developments in religious, ideological, and political structures in a distinct population.

## Conclusion

These articles go a long way toward identifying evolutionary studies in imaginative culture as a distinct field. They also suggest ways in which the field could be more fully integrated and conceptually coherent. Flinn's outline of human life history offers a sound evolutionary framework for life phases and social and reproductive relationships, but other articles rightly point toward adaptive functions of imagination that are not exclusively social. A more complete understanding of the adaptive functions of imagination can be derived from research on the DMN. Research on the DMN can and should replace speculative and impressionistic views of the imagination with a precise and illuminating set of neurological facts. Most of the contributors to this Research Topic seem to register, even if only casually, how important emotion is to imaginative experience. The next step for many scholars and scientists should be to inform themselves about the most advanced research in emotion theory—findings and theory that go far beyond Ekman's ideas about “basic emotions” or Panksepp's ideas about conserved mammalian neurological systems (Cowen et al., 2019; Carroll, 2022). Life history theory can provide a framework for basic subjects or themes in literature; emotion theory can provide a framework for emotional tone; and cognitive science can provide a framework for formal structures (Carroll, 2018). Accordingly, Flinn's work on life history, McCrae's work on emotions in music, and Kozbelt's work integrating evolved cognitive dispositions with formal aesthetic structures can help point the way toward a unified conception of imaginative artifacts. That unified conception is a necessary prerequisite to a unified understanding of imaginative culture.

## Author contributions

All authors listed have made a substantial, direct, and intellectual contribution to the work and approved it for publication.

## Conflict of interest

The authors declare that the research was conducted in the absence of any commercial or financial relationships that could be construed as a potential conflict of interest.

## Publisher's note

All claims expressed in this article are solely those of the authors and do not necessarily represent those of their affiliated organizations, or those of the publisher, the editors and the reviewers. Any product that may be evaluated in this article, or claim that may be made by its manufacturer, is not guaranteed or endorsed by the publisher.
